# Epidemiological trends in TB during a technical assistance project, Zambia, 2015–2018

**DOI:** 10.5588/pha.24.0028

**Published:** 2024-12-01

**Authors:** M. Ota, V. Mfungwe, C. Masitano, Y. Matsuoka, S. Hirao, S. Amano, Y. Tanaka, S. Daka, M. Oniki-Goto, M. Phiri, Z. Mtonga, M. Changala, G.K.V. Samungole, C.Y. Msiska

**Affiliations:** ^1^ Research Institute of Tuberculosis, Japan Anti-Tuberculosis Association (JATA), Tokyo, Japan;; ^2^ JATA Zambia, Lusaka, Zambia;; ^3^ International Programmes, JATA, Tokyo, Japan;; ^4^ Chongwe District Health Office, and; ^5^ National Tuberculosis and Leprosy Programme, Ministry of Health, Republic of Zambia, Lusaka, Zambia.

**Keywords:** epidemiology, tuberculosis, Chongwe

## Abstract

**SETTING:**

Three health facilities: Chongwe Health Centre (CHC), Chongwe District Hospital (CDH), and Ngwerere Health Centre (NHC) in Chongwe District, Lusaka Province, Zambia.

**OBJECTIVE:**

To describe the epidemiological trend of TB in 2015–2018, with the 2014 data as baseline.

**DESIGN:**

This was an observational study.

**RESULTS:**

At CHC, CDH, and NHC, 457, 851 and 85 cases, respectively, of all types of TB were registered in 2014–2018. The numbers of patients with presumptive TB at CHC and CDH increased from 606 and 406, respectively, in 2014 to 1068 and 1848, respectively, in 2018. The proportion of patients with bacteriologically positive TB among patients with presumptive TB decreased at CHC and CDH from over 10% in 2014 to less than 5% in late 2018. The treatment success rates decreased at CHC and CDH in 2017 from respectively 93.7% and 93.0% in 2014 to 69.1% and 73.0% in 2017. GeneXpert equipment was installed at CHC in 2016 and CDH in 2017.

**CONCLUSION:**

After introducing GeneXpert equipment at CHC and CDH, the proportion of bacteriologically-positive TB among presumptive TB significantly decreased because of the high number of patients with presumptive TB screened; however, it may have also caused treatment success rates to have stumbled.

## Abstract (fr)

**CONTEXTE:**

Trois établissements de santé : Centre de santé de Chongwe (CHC), Hôpital du district de Chongwe (CDH) et Centre de santé de Ngwerere (NHC) situés dans le district de Chongwe, province de Lusaka, Zambie.

**OBJECTIF:**

Décrire la tendance épidémiologique de la TB entre 2015 et 2018, en utilisant les données de 2014 comme point de comparaison.

**MÉTHODE:**

Il s'agit d'une étude basée sur l'observation.

**RÉSULTATS:**

Au CHC, au CDH et au NHC, un total de 457, 851 et 85 cas de TB de tous types a été enregistré entre 2014 et 2018. Le nombre de patients présentant une TB présumée au CHC et au CDH a augmenté, passant de 606 et 406 en 2014 à 1 068 et 1 848 en 2018, respectivement. La proportion de patients ayant une TB bactériologiquement positive parmi ceux ayant une TB présumée a connu une diminution au CHC et au CDH, passant de plus de 10% en 2014 à moins de 5% à la fin de 2018. Les taux de réussite des traitements ont également baissé au CHC et au CDH en 2017, chutant respectivement de 93,7% et 93,0% en 2014 à 69,1% et 73,0% en 2017. L'équipement GeneXpert a été mis en place au CHC en 2016 et au CDH en 2017.

**CONCLUSION:**

Après l'introduction de l'équipement GeneXpert au CHC et au CDH, la proportion de TB bactériologiquement positives parmi les cas présumés a considérablement baissé, grâce à un nombre accru de patients suspectés de TB ayant été dépistés. Néanmoins, cette situation a également pu affecter les taux de réussite du traitement.

The Republic of Zambia (hereafter, Zambia) is one of the 30 countries with a high burden of TB, with an incidence rate of 361/100,000 population in 2017 estimated by the WHO.^1^ The high prevalence of HIV infection, which was 12.2% among those aged from 15 to 49 years in 2017, estimated by the Joint United Nations Programme on HIV/AIDS (UNAIDS), prompted the epidemic.^2^ The burden of TB is exceptionally high in Lusaka province, where two-fifths of the patients with TB in the country are accumulated.

We previously carried out a 3-year TB control project in urban areas of Lusaka District to mitigate the problems concerning TB and HIV, resulting in an increased number of patients with presumptive TB examined at the government health facilities and improved treatment outcomes with the help of community health volunteers (CHVs).^3–5^ We then moved to a more rural area^6^ and tried to apply the experiences we obtained in Lusaka and elsewhere^7–15^ to strengthen case-finding and management of patients with TB in Chongwe District, Lusaka Province, Zambia, where the number of TB diagnostic centres is limited, and it often takes hours to access to health facilities.

This report aims to describe the epidemiological trend of patients with TB at three health facilities in Chongwe District, Lusaka Province, Zambia, from 2015 to 2018, using the data from 2014 as the baseline.

## Study population, design and method

**FIGURE 1.**
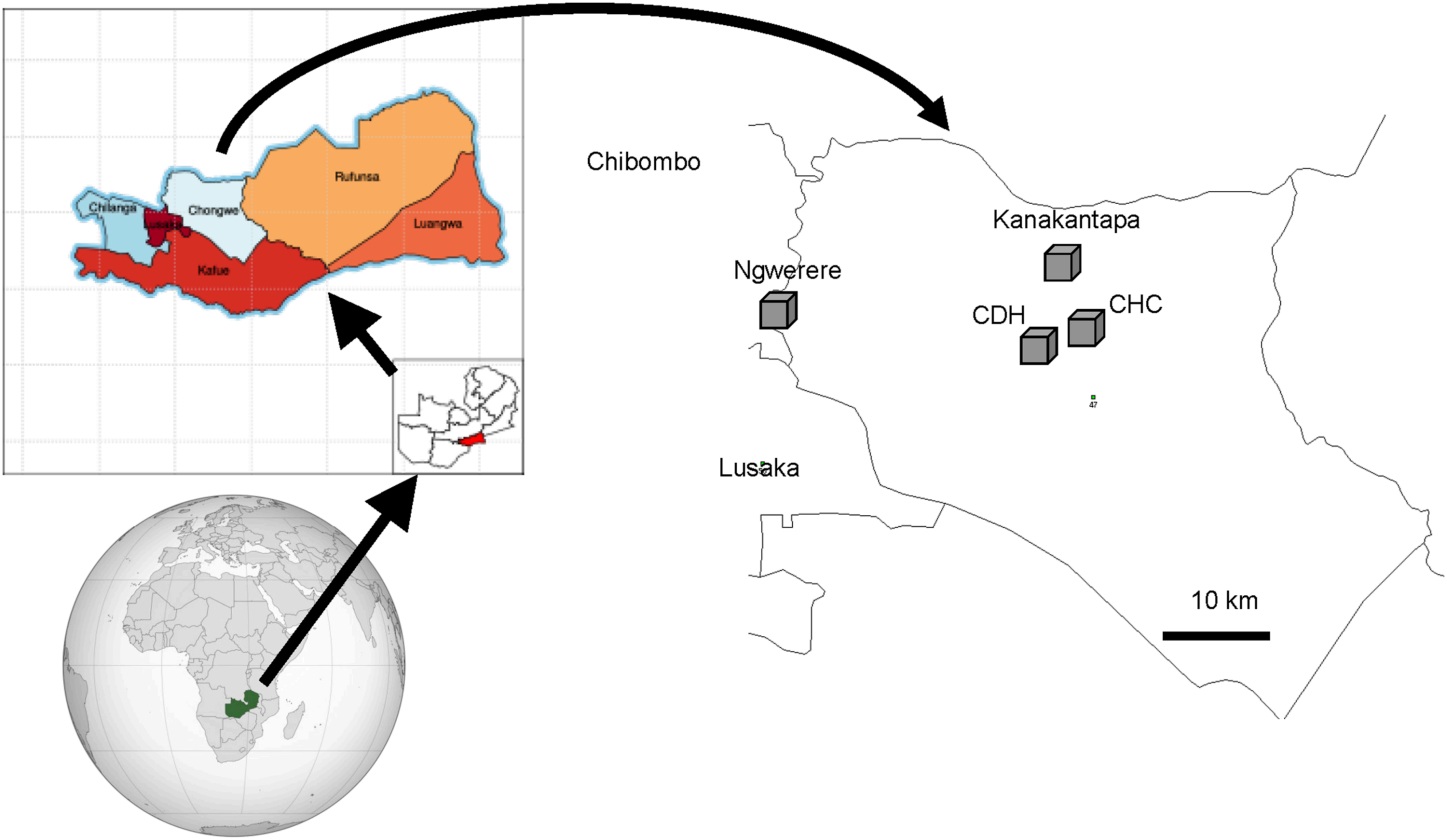
Locations of healthcare facilities in Chongwe District, Zambia (maps were derived from Wikipedia). CHC = Chongwe Health Centre; CDH = Chongwe District Hospital.

Chongwe is located in central Zambia (Figure 1), with a population of 141,000 in 2010.^16^ It borders Lusaka, the capital city of Zambia, to the west. There were three TB diagnostic centres within Chongwe, one of which was a military hospital that ordinary people could not access. The remaining two, Chongwe Health Centre (CHC) and Chongwe District Hospital (CDH) were initially chosen as the project sites. Two others, the Kanakantapa and Ngwerere Health Centres (HCs), were added later. CHC is in the central part of the district on the Great East Road, connecting Lusaka and Lilongwe, the capital city of Malawi. It used to be a referral health centre before 2014 and had a laboratory with a GeneXpert machine (Cepheid, Sunnyvale, CA, USA), which can detect TB bacilli as well as rifampicin resistance from a sputum sample at the same time*,* installed in 2016. CDH is located 1.5 km west of CHC on the junction toward Lusaka and Chirundu, a town bordering Zimbabwe. CDH had an X-ray machine and a laboratory with a GeneXpert machine installed in 2017. Kanakantapa HC (KHC) is 6 km north northwest of CHC, and Ngwerere HC (NHC) is 40 km west of CHC, bordering Lusaka and Chibombo districts.

According to the National TB and Leprosy Programme (NTLP) of Zambia,^17^ the diagnosis of TB is made based on bacteriological confirmation or clinical presentation. Once a patient with presumptive TB attends an outpatient department (OPD) of a health facility, a medical or clinical officer asks the patient to submit a sputum sample on the spot to the laboratory to evaluate the patient bacteriologically. The sputum sample is examined using GeneXpert. If a GeneXpert is unavailable at the laboratory, two sputum samples are usually requested and examined with smear microscopy. Suppose a negative result is obtained, but a clinician still suspects TB. In that case, the patient is sent to the X-ray department to evaluate the lungs and radiological and clinical evaluation is used to guide the diagnosis. If the sputum sample is positive for TB bacilli, or the medical or clinical officer believes that the patient has TB, the patient is registered, and they initiate anti-TB treatment with isoniazid, rifampicin, pyrazinamide, and ethambutol for 2 months and then isoniazid and rifampicin for 4 months. During the anti-TB treatment, the patient is seen at least once a month by a healthcare provider for clinical review and assessment of side effects. In addition, the patient is asked to submit sputum samples to the laboratory at the end of Months 2 and 4 and during Month 6 to monitor the treatment progress. The samples are examined with sputum smear microscopy using the Ziehl-Nielsen method.

The project conducted four main activities to improve case-finding and treatment outcomes of TB: 1) training of CHVs and healthcare workers (HCWs) concerning TB control, 2) organising CHV activities on sensitisation about TB and HIV in the community, 3) monitoring visits to the health facilities regarding the management of patients with TB, and 4) holding biannual review meetings on TB control for the health facilities in Chongwe. The training included basic training for the CHVs, training of trainers (TOT) for the TB outpatient clinics (TB corners) nurses, training on taking chest X-ray (CXR) for radiology technicians, and training on reading CXR for medical and clinical officers. The CHVs paid visits to the homes of patients with TB and held community sensitisation sessions bimonthly in the community. To monitor the case finding and treatment outcomes of the health facilities, the project staff members conducted quarterly visits to the TB corners and the laboratories, collecting data from the TB registers, the laboratory and the presumptive TB registers. Semiannual review meetings were held to discuss issues related to TB control in the four areas and the project’s activities and achievements.

We reviewed the numbers and trends of cases with TB, including all types and bacteriologically positive (bac+) TB and presumptive TB, and treatment outcomes, including treatment success rates and rates of loss to follow-up (LTFU) at the three health facilities in Chongwe District mentioned above, excluding KHC because the number of patients with TB registered there was small: less than five each year. Since TB treatment needs to continue for 6 months and the data are typically reviewed 1 year after the initiation of the treatment, we examined the treatment outcomes of the patients registered in 2015–2017 with the 2014 data as the baseline. The definitions for types of TB disease and treatment outcomes were defined according to the WHO guideline.^18^

Statistical tests were conducted using R x64 v4.0.2 (The R Foundation for Statistical Computing, Vienna, Austria). Spearman’s correlation test was employed to determine the trend of the quarterly numbers of TB cases. The Cochran-Armitage test was used to determine the trends of treatment success and LTFU rates. *P* < 0.05 was considered statistically significant.

The study was conducted as a part of the routine evaluation activities of a project called “Comprehensive TB/HIV Control with a Strengthened Community Participation in Chongwe District” with the Memorandum of Understanding cosigned by the Ministry of Health of the Republic of Zambia, the Chongwe District Health Office, and the Japan Anti-Tuberculosis Association in February 2015. In compiling the data on case-finding and treatment outcomes of patients with TB, the names and addresses of the patients were not collected to protect their confidentiality.

The authors obtained a waiver of institutional review for conducting the study from the institutional review board of the Ethics in Research Ethics and Science Converge, Lusaka, Zambia (Ref. No: 2024-Feb-029). We also obtained permission from the National Health Research Authority of Zambia, Lusaka, Zambia, to publish the study (Ref No: NHRA 1058/19/3/2024).

## RESULTS

Initially, two facilities, CHC and CDH, were the main TB diagnostic centres in Chongwe. From around 2016 to late 2017, GeneXpert and X-ray equipment were installed at NHC, establishing it as one of the TB diagnostic centres in Chongwe by the end of 2017. At KHC, smear microscopy was only intermittently available because of a staffing shortage at the laboratory, and the number of patients with TB registered there was continuously less than five per year; thus, we excluded the HC from this report as described in Methods.

**FIGURE 2.**
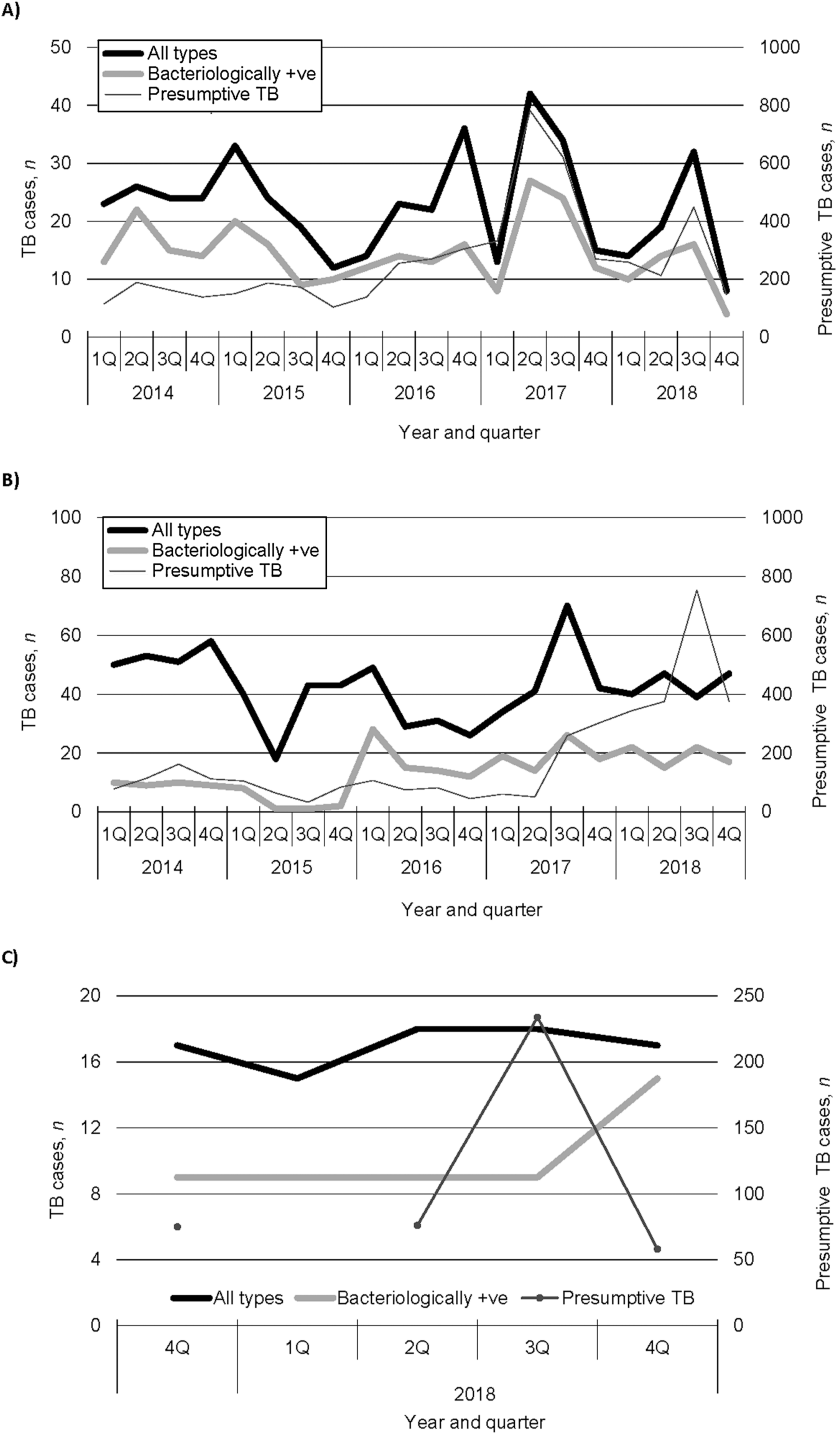
The numbers of the presumptive, all types of, and bacteriologically positive TB patients at **1)** CHC, **2)** CDH, and **C)** Ngwerere Health Centre, Chongwe, Zambia, 2014–2018. **A)** At CHC, the numbers of all types of and bac+ TB exhibited neither increasing nor decreasing trends (ρ = –0.22, *P* = 0.34 and ρ = –0.19, *P* = 0.41, respectively), whereas the number of the presumptive TB cases showed a statistically significant increasing trend (ρ = 0.57, *P* = 0.01). **B)** At CDH, the number of all types of TB exhibited neither an increasing nor a decreasing trend (ρ = –0.26, *P* = 0.27), whereas the numbers of bacteriologically +ve and presumptive TB cases showed increasing trends (ρ = 0.70, *P* = 0.0001 and ρ =0.44, *P* < 0.05, respectively). **C)** At Ngwerere HC, the numbers of all types of and bacteriologically +ve TB did not have either increasing or decreasing trends (ρ = 0.37, *P* = 0.54 and ρ = 0.71, *P* = 0.18, respectively). +ve = positive; CHC = Chongwe Health Centre; CDH = Chongwe District Hospital.

**FIGURE 3.**
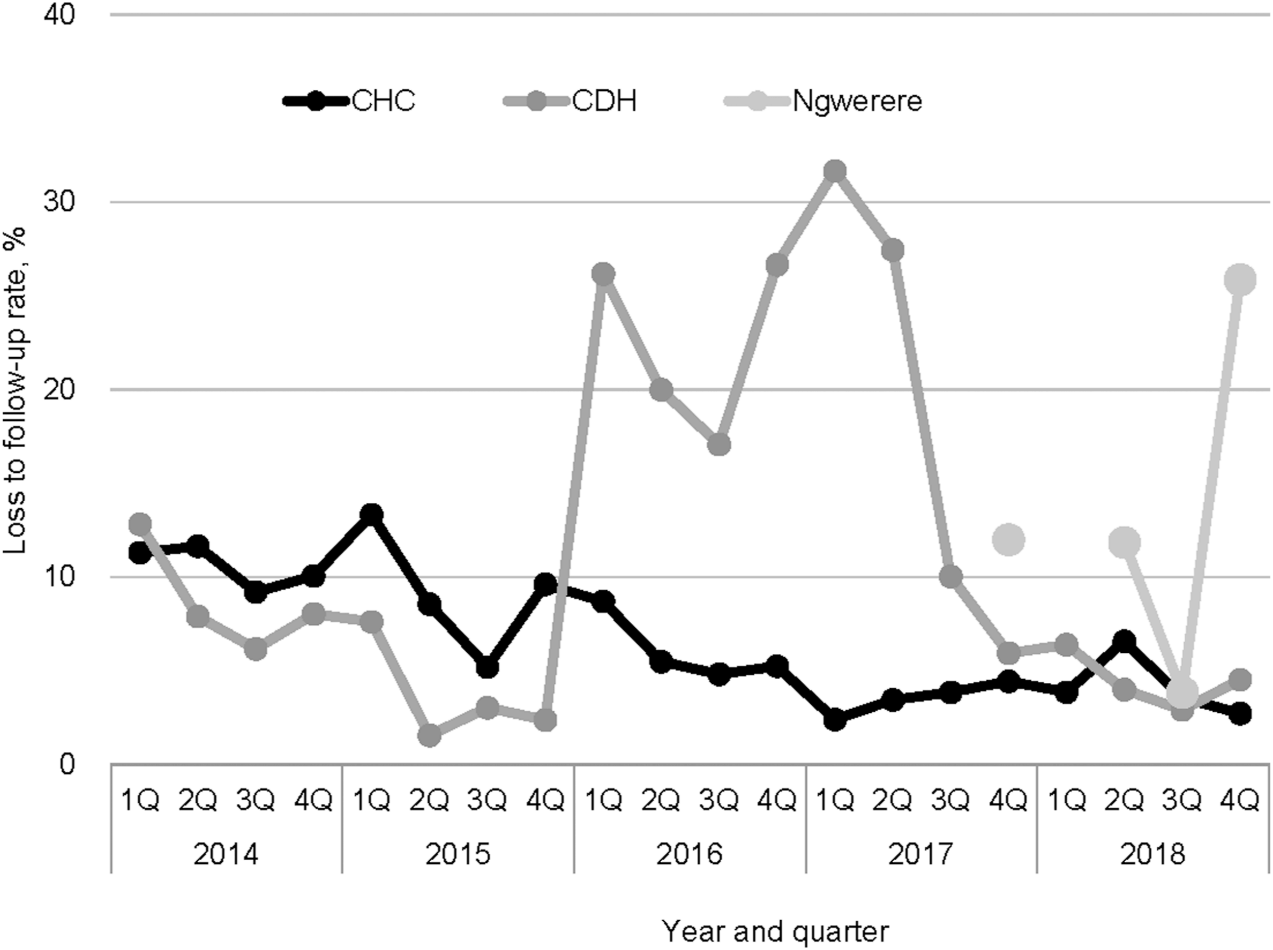
The proportions of patients with actual TB among presumptive ones by health facility, Chongwe, Zambia, 2014–2018. CHC = Chongwe Health Centre; CDH = Chongwe District Hospital; Q = quarter.

The Table shows the number of cases of TB found from 2014 to 2018 by the facilities. CDH had the highest number of cases of TB, followed by CHC and NHC. Figure 2 shows the trends of case finding at the three TB diagnostic centres in Chongwe from 2014 to 2018 regarding all types of TB, including bac+ and presumptive TB. At CHC, the numbers of all types of and bac+ TB exhibited neither increasing nor decreasing trends (Figure 2A) (ρ = –0.22, *P* = 0.34 and ρ = –0.19, *P* = 0.41, respectively), whereas the number of cases with presumptive TB showed a statistically significant increasing trend (ρ = 0.57, *P* = 0.01). At CDH, the number of all types of TB exhibited neither an increasing nor a decreasing trend (Figure 2B) (ρ = –0.26, *P* = 0.27), whereas the numbers of cases with bac+ and presumptive TB showed increasing trends (ρ = 0.70, *P* = 0.0001 and ρ = 0.44, *P* < 0.05, respectively). At NHC, the numbers of all types of TB and bac+ TB did not have an increasing or decreasing trend (Figure 2C) (ρ = 0.37, *P* = 0.54 and ρ = 0.71, *P* = 0.18, respectively). The trend could not be calculated for the number of cases with presumptive TB because there was a missing value during the study period. The trends for the proportions of bac+ TB cases among the presumptive TB cases were also calculated, and they were decreasing at both CHC and CDH (both *P* < 0.0001), whereas at NHC, the trend could not be calculated because of missing values in Q1 of 2018 (Figure 3).

**TABLE.** Numbers of cases of TB by facility, Chongwe, Zambia, 2014–2018.

	All types of TB	Bacteriologically positive TB	Presumptive TB
	*n* (%)	*n* (%)	*n* (%)
Chongwe Health Centre	457 (8.7)	289 (5.5)	5,262 (100)
Chongwe District Hospital	851 (23.8)	272 (7.6)	3,582 (100)
Ngwerere Health Centre*	85 (19.2)	51 (11.5)	443 (100)
Total	1,393 (15.0)	612 (6.6)	9,287 (100)

* Ngwerere Health Centre started to serve as a diagnostic centre in Q4 of 2017 and its figures include only those for five quarters in 2017–2018.

**FIGURE 4.**
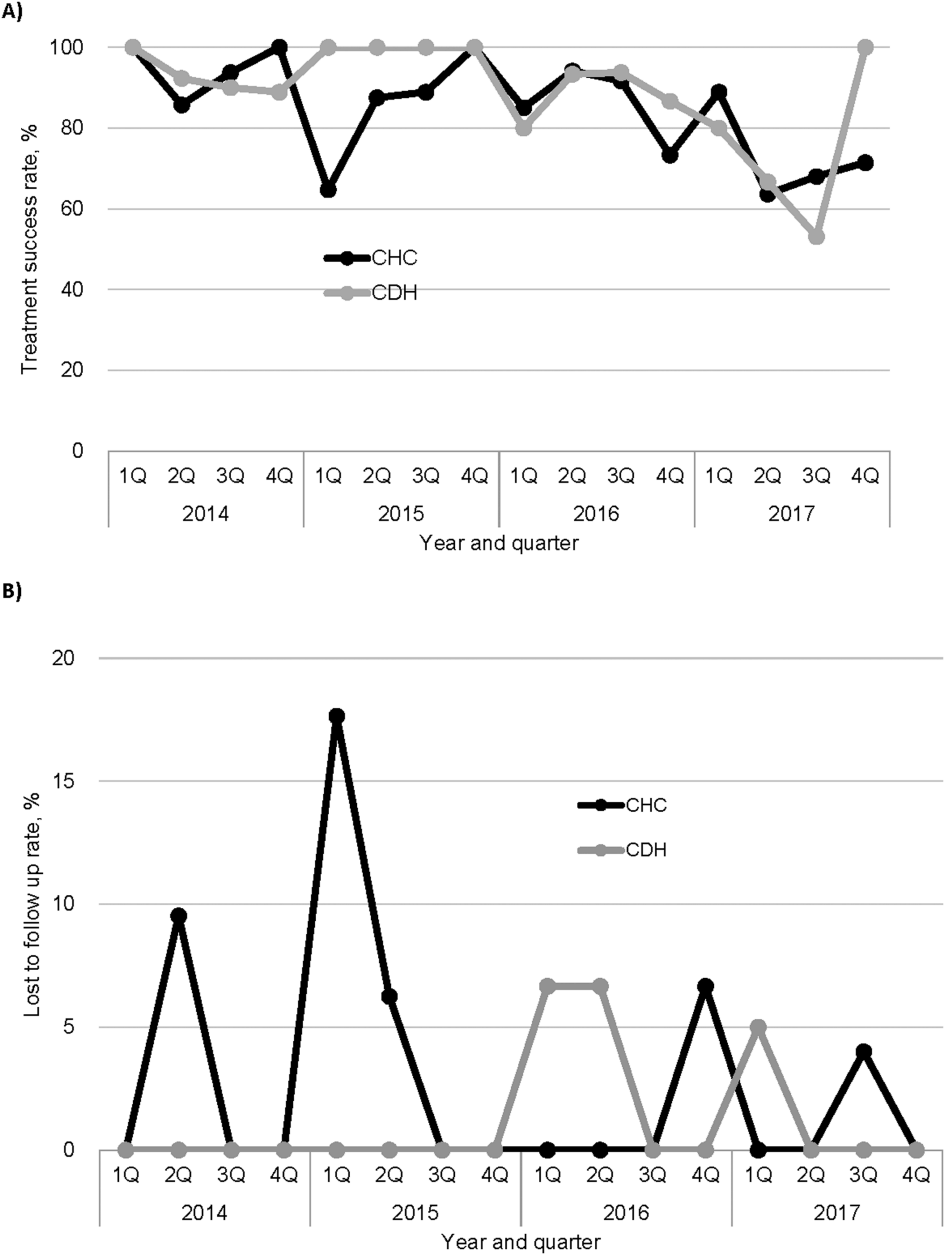
**A)** Treatment success, and **B)** loss to follow-up rates of patients with TB by a health facility, Chongwe, Zambia, 2014–2017. As treatment outcomes were reviewed 1 year after the patients initiated the treatment, data were available only up to 2017. CHC = Chongwe Health Centre; CDH = Chongwe District Hospital.

Figure 4 shows the treatment success and LTFU rates of CHC and CDH for the bac+ TB patients registered from 2014 through 2017. The treatment success rates significantly decreased at CHC and CDH toward 2017 (*P* < 0.0001 and *P* = 0.003, respectively), whereas the LTFU rates did not change substantially at CHC and CDH (*P* = 0.14 and *P* = 0.99, respectively). At NHC in Q4 of 2017, the treatment success rate was 87.5%, and the LTFU rate was 12.5%. The rates of ‘not evaluated’, previously known as ‘transferred out’ increased both in CHC and CDH from Q4 of 2016 toward the end of 2017, ranging between 5% and 40%, with peaks at 28.0% at CHC and 43.8% at CDH in Q3 of 2017.

A total of 60 CHVs were recruited with recommendations from TB corner nurses: 15 each from CHC, CDH, KHC, and NHC. They were trained and mobilised to conduct community sensitisation activities, support patients with TB at the TB corner of each health facility, and visit them at home. In total, 3573 community members attended bimonthly sensitisation activities organised by the CHVs in 2016, 15,755 in 2017, and 96,878 in 2018.

The project supported four types of training: 1) training of trainers (TOT) for the TB corner and HIV/AIDS clinic nurses of the four facilities, 2) for the radiology technicians at the CDH and later Ngwerere, 3) for the laboratory technologists, and 4) for the medical and clinical officers. Training courses on CXR examinations and reading CXR films were held in July and October 2018, respectively. Training sessions regarding fluorescence smear microscopy were held for laboratory technologists in July 2018.

The project staff members conducted quarterly monitoring visits to the TB corners. A total of three quarterly meetings were held with attendance from 23 health facilities in Chongwe, the staff members of the TB corners, and the representatives of the CHVs and the project in 2018.

## DISCUSSION

The authors reviewed the epidemiology of TB in terms of case-finding and treatment outcomes in areas under three health facilities in the Chongwe district. We noted that the introduction of GeneXpert machines with higher sensitivity than smear microscopy at CHC and CDH resulted in them finding large numbers of patients with presumptive TB, leading to a decrease in the proportions of bac+ TB among the patients with presumptive TB from over 10% in early 2014 to less than 5% at the end of 2018. This finding suggests that the burden of TB in the community may decrease.^5,19,20^ However, because some patients were from outside their catchment areas and the district, there were significant ‘not evaluated’ rates at both clinics. In addition, although it is not apparent from our current study, from our previous experience, we should be careful about patients with presumptive TB from outside of catchment areas because they may not come back to commence their anti-TB treatment (so-called pre-treatment lost to follow-up) after they were found to have TB.^6,21^

The LTFU rates improved at CHC and, particularly, CDH because the CHVs made great efforts to support the patients with TB and encouraged them to complete their treatment. In addition, the TB corner nurses had become more committed to following up with patients with TB after the series of training sessions and supportive visits to the clinics, as we have seen before in the Lusaka District.^5^

The findings of this report are in line with past studies on strengthening case findings in Haiti, Ethiopia, and other areas, including our own,^5,18,22–24^ in which the provision of training for HCWs and CHVs helped increase the number of patients with presumptive TB examined at the clinics and then also the number of patients with TB. However, though the introduction of GeneXpert machines may increase the number of patients found with bac+ TB, it did not necessarily increase the number of patients with all types of TB found in eastern Nepal,^25^ as was seen at CHC and CDH in our study. In Flores, Indonesia, it was reported that improving knowledge about the cause, transmission and prevention of TB led to earlier case detection^26^ and the activities of CHVs in our study, particularly the bimonthly community sensitisation concerning TB and HIV/AIDS, may have contributed to a better understanding of TB and possibly decreased its burden in the community.

Laboratories played an important role in diagnosing and monitoring patients with TB and in the surveillance system to monitor resistance to anti-TB drugs, as GeneXpert machines can detect rifampicin resistance from sputum. As such, the project provided some laboratory equipment, including GeneXpert and air conditioners, to the laboratory rooms and provided training to the laboratory technologists. We therefore recommend that every project supporting NTPs should include laboratory components.^5^

There are some limitations in this study. One of them was that this was a technical assistance project on strengthening TB control, and it is unclear whether the results of the review can be extrapolated to other places; however, it is still useful for those who will run similar TB control projects in countries with a high-burden of TB, particularly in sub-Saharan Africa where the health workforce is limited, and the prevalence of HIV is high among the general population.

In conclusion, the introduction of GeneXpert, which has higher sensitivity than conventional smear microscopy, may reduce the burden of TB in the community. Based on this and past studies on strengthening TB control in rural settings, we recommend that ministries of health, NTPs and district health offices enhance the management of patients with TB at clinics to improve treatment outcomes with the help of CHVs.

## References

[1] World Health Organization. Global tuberculosis report, 2018. Geneva, Switzerland: WHO, 2018.

[2] UNAIDS. HIV epidemiological estimates spreadsheet. Geneva, Switzerland: UNAIDS, 2022.

[3] Mfungwe V, ‘Transfer out’ tuberculosis patients: treatment outcomes after cross-checking registers, 2012–2013, Lusaka, Zambia. Public Health Action. 2016;6(2):118–121. 27358805 10.5588/pha.16.0016 PMC4913674

[4] Toyama Y, Which community volunteers participate most frequently in support programs for TB patients? Case report from Lusaka, Zambia, 2015. J Int Health. 2020;35(2):113–120.

[5] Ota M, Experience of a technical cooperation project on strengthening a local national tuberculosis programme, Lusaka, Zambia: 2012–2015. J Int Health. 2021;36(4):195–202.

[6] Chilembo M, Pre-treatment lost to follow-up tuberculosis patients, Chongwe, Zambia, 2017: a retrospective cohort study. Public Health Action. 2020;10(1):21–26. 32368520 10.5588/pha.19.0059 PMC7181362

[7] Toyama Y, Event-based surveillance in north-western Ethiopia: experience and lessons learnt in the field. Western Pac Surveill Response J. 2015;6(3):22–27. 10.5365/WPSAR.2015.6.2.002 PMC4675161 26668763

[8] Toyama Y, Sharp decline of malaria cases in the Burie Zuria, Dembia, and Mecha districts, Amhara Region, Ethiopia, 2012–2014: descriptive analysis of surveillance data. Malar J. 2016;15:104. 26892875 10.1186/s12936-016-1133-9 PMC4759934

[9] Ota M, Strengthening the communicable disease surveillance and response system, Amhara Region, Ethiopia, 2012–2014: review of a technical cooperation project. J Int Health. 2017;32(1):1–8.

[10] Mao TE, Cross-sectional studies of tuberculosis prevalence in Cambodia between 2002 and 2011. Bull World Health Org. 2014;92(8):573–581. 25177072 10.2471/BLT.13.131581 PMC4147411

[11] Nagata Y, Difficulty of confining recalcitrant tuberculosis patients in isolation wards in Japan, 2013–2014. Public Health. 2017;154:31–36. 29169073 10.1016/j.puhe.2017.10.010

[12] Urakawa M, Tuberculosis-related technical enquiries received by a national-level institution in Japan, 2014–2016. Public Health Action. 2018;8(3):130–134. 30271729 10.5588/pha.18.0034 PMC6147068

[13] Urakawa M, TB-related technical enquiries received in Japan, 2017–2019. Public Health Action. 2022;12(4):206–209. 36561899 10.5588/pha.22.0053 PMC9716821

[14] Ota M, Trends of tuberculosis rates before and after the declaration as a public health emergency in Japan, 1992–2006. Int J Tuberc Lung Dis. 2019;23(9):1000–1004. 31615607 10.5588/ijtld.18.0650

[15] Ota M, Lower TB notification rates in later life in the same birth cohort, Japan, 1950–2020. Int J Tuberc Lung Dis. 2024;28(3):157–159. 38454190 10.5588/ijtld.23.0092

[16] Zambia Statistics Agency. 2022 Census of population and housing: preliminary report. Zambia Statistics Agency. Lusaka, Zambia: ZSA, 2022.

[17] National Tuberculosis and Leprosy Programme, Ministry of Health of the Republic of Zambia. TB Manual. 5^th^ ed. Ministry of Health of the Republic of Zambia, 2017.

[18] World Health Organization. Definitions and reporting framework for tuberculosis, 2013 revision. Geneva, Switzerland: WHO, 2013.

[19] Ayles H, Effect of household and community interventions on the burden of tuberculosis in southern Africa: the ZAMSTAR community-randomised trial. Lancet. 2013;382:1183–1194. 23915882 10.1016/S0140-6736(13)61131-9

[20] Nugussie DA, Prevalence of smear-positive tuberculosis among patients who visited Saint Paul’s specialised hospital in Addis Ababa, Ethiopia. Biomed Res Int. 2017;2017:6325484. 28904965 10.1155/2017/6325484 PMC5585560

[21] Daka S, Causes of pre-treatment loss to follow-up in patients with TB. Public Health Action. 2022;12(4):148–152. 36561903 10.5588/pha.22.0051 PMC9716822

[22] Charles M, Trends in tuberculosis case notification and treatment success, Haiti, 2010–2015. Am J Trop Med Hyg. 2017;97(Suppl 4):49–56. 10.4269/ajtmh.16-0863 PMC5676628 29064365

[23] Dememew ZG, Trends in tuberculosis case notification and treatment outcomes after interventions in 10 zones of Ethiopia. Int J Tuberc Lung Dis. 2016;20(9):1192–1198. 27510245 10.5588/ijtld.16.0005

[24] Creswell J, A multi-site evaluation of innovative approaches to increase tuberculosis case notifications: summary results. PLoS One. 2014;9(4):e94465. 24722399 10.1371/journal.pone.0094465 PMC3983196

[25] Creswell J, Introducing new tuberculosis diagnostics: the impact of Xpert(R) MTB/RIF testing on case notifications in Nepal. Int J Tuberc Lung Dis. 2015;19(5):545–551. 25868022 10.5588/ijtld.14.0775

[26] Dewi C, Improving knowledge and behaviours related to the cause, transmission and prevention of tuberculosis and early case detection: a descriptive study of community-led tuberculosis program in Flores, Indonesia. BMC Public Health. 2016;16:740. 27503095 10.1186/s12889-016-3448-4 PMC4977733

